# BioMimics 3D Stent in Femoropopliteal Lesions: 3-Year Outcomes with Propensity Matching for Drug-Coated Balloons

**DOI:** 10.3390/jcdd10030126

**Published:** 2023-03-16

**Authors:** Michael Piorkowski, Thomas Zeller, Christos Rammos, Koen Deloose, Klaus Hertting, Volker Sesselmann, Gunnar Tepe, Peter Gaines, Michael Lichtenberg

**Affiliations:** 1 Department of Vascular Medicine, Cardioangiologic Center Bethanien, 60389 Frankfurt, Germany; 2 Department of Angiology, Universitätsklinikum Freiburg Herzzentrum, 79189 Bad Krozingen, Germany; 3 Department of Cardiology and Vascular Medicine, West German Heart and Vascular Center Essen, University Hospital Essen, 45147 Essen, Germany; 4 Department Vascular Surgery, AZ Sint-Blasius Dendermonde, Kroonveldlaan 50, 9200 Dendermonde, Belgium; 5 Department of Cardiology and Angiology, Krankenhaus Buchholz und Winsen gGmbH, 21423 Buchholz, Germany; 6 Department of Angiology, SRH Zentralklinikum Suhl GmbH, 98527 Suhl, Germany; 7 Department of Diagnostic and Interventional Radiology, RoMed Klinikum Rosenheim, 83022 Rosenheim, Germany; 8 College of Health, Wellbeing and Life Sciences, Sheffield Hallam University, Sheffield S1 1WB, UK; 9 Vascular Center, Klinikum Hochsauerland, 59821 Arnsberg, Germany

**Keywords:** peripheral artery disease, stent, drug-coated balloon

## Abstract

Background: Through its helical centreline geometry, the BioMimics 3D vascular stent system is designed for the mobile femoropopliteal region, aiming to improve long-term patency and the risk of stent fractures. Methods: MIMICS 3D is a prospective, European, multi-centre, observational registry to evaluate the BioMimics 3D stent in a real-world population through 3 years. A propensity-matched comparison was performed to investigate the effect of the additional use of drug-coated balloons (DCB). Results: The MIMICS 3D registry enrolled 507 patients (518 lesion, length 125.9 ± 91.0 mm). At 3 years, the overall survival was 85.2%, freedom from major amputation 98.5%, freedom from clinically driven target lesion revascularisation 78.0%, and primary patency 70.2%. The propensity-matched cohort included 195 patients in each cohort. At 3-year follow-up, there was no statistically significant difference in clinical outcomes, such as overall survival (87.9% in the DCB vs. 85.1% in the no DCB group), freedom from major amputation (99.4% vs. 97.2%), clinically driven TLR (76.4% vs. 80.3%), and primary patency (68.5% vs. 74.4%). Conclusion: The MIMICS 3D registry showed good 3-year outcomes of the BioMimics 3D stent in femoropopliteal lesions, demonstrating the safety and performance of this device under real-world conditions, whether used alone or in combination with a DCB.

## 1. Introduction

Successful procedural outcomes and long-term durability are the main goals in the interventional treatment of peripheral artery disease (PAD), especially in the femoropopliteal lesions. Endovascular treatment options have substantially improved over the last decades, but there is still room for enhancements, such as improvement in long-term patency and durability of stents in this highly mobile region [1].

The BioMimics 3D vascular stent system (Veryan Medical Ltd., Horsham, UK) features a new-generation self-expanding nitinol stent, with a 3D helical centreline. It is designed to resist forces such as compression, bending and torsion of the artery, and aims to improve patency rates compared to other bare metal stents by mimicking natural structures [2,3]. In the MIMICS randomised controlled trial, the BioMimics 3D stent was superior to straight stents [4]; the MIMICS 3D all-comers registry aims to assess the safety and performance of the BioMimics 3D stent in a real-world setting, leaving the additional use of drug-coated balloons (DCB) at the discretion of the operators. Data up to 2 years have been reported previously [2]; we herein report final data out to 3 years.

To further elucidate the effect of DCB combined with stent treatment, a propensity-score-matched analysis was performed.

## 2. Materials and Methods

### 2.1. Study Design

The prospective, multi-centre, observational registry has been described in detail previously [2]. In brief, the registry aimed to evaluate the safety, effectiveness and performance of the BioMimics 3D self-expanding stent system in a real-world population. Patients were enrolled in 23 centres in Europe between September 2016 and June 2018. Follow-up visits were planned at 30 days, and at 12, 24 and 36 months according to centres’ standard of care. This is the final report including 3-year data.

Inclusion criteria were adult patients ≤85 years of age, written informed consent, and symptomatic PAD of femoropopliteal lesions, scheduled for treatment with the BioMimics 3D stent according to the instructions for use. Exclusion criteria were inability to cross the lesion with a wire or balloon catheter, intolerance to antiplatelet and/or anticoagulation therapy, bleeding diathesis, severe hypertension, renal failure, known hypersensitivity to nickel–titanium, comorbidity that limits the life expectancy to less than 12 months, pregnant or breastfeeding patients, or patients that were unwilling to comply with the standard of care procedures and follow-up visit. 

An independent clinical events committee was responsible for the systematic review and adjudication of all major adverse events, including death and all potentially device-related adverse events. The registry complies with the current version of the Declaration of Helsinki, relevant data protection regulations, international standards, and regulations applicable to medical device registries. It was approved by the respective ethics committees and each patient provided written informed consent. The registry is listed on www.clinicaltrials.gov (NCT02900924).

### 2.2. Study Device and Procedure

**Figure 1:**
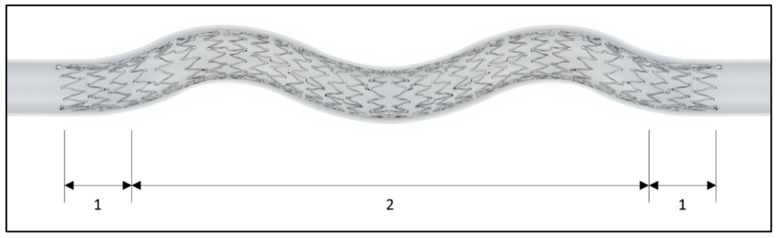
Unconstrained BioMimics 3D stent. The BioMimics 3D stent has a helical centreline (2) and transition sections (1). The last 3 crowns at the stent ends define the transition sections, where the length of the crown is increased, slightly reducing the radial force imposed on the vessel, thus mitigating a step-change in the profile at the stent ends. Image provided by Veryan Medical.

The BioMimics 3D stent system consists of a self-expanding nitinol stent, with three-dimensional helical centreline geometry stored in the nitinol-shape memory (Figure 1), and a 6F over-the-wire delivery system. The stent is available in diameters of 5, 6, and 7 mm, and lengths of 60 to 150 mm. Additional DCB use was left at the investigators’ discretion. If the physician decided that no treatment with the BioMimics 3D stent was necessary during the procedure, the patient was not considered to be enrolled in the registry.

### 2.3. Endpoints and Definitions

Endpoints at 36 months are death, target limb amputation, clinically driven target lesion revascularisation (CD-TLR), primary patency (site-assessed, defined as peak systolic velocity ratio <2.4 by duplex ultrasound), and functional and clinical outcomes, including ankle–brachial index (ABI) and Rutherford class.

### 2.4. Statistical Analysis

The analysis is based on the intention-to-treat population. Continuous variables are expressed as the mean ± standard deviations, categorical variables are expressed as frequencies. All calculations are based on the data available. The *p*-values were calculated using a Fisher’s exact test for categorical variables, Student’s *t*-test (means) for continuous variables and log-rank test for comparing Kaplan–Meier estimates. A *p*-value < 0.05 was considered significant.

A subgroup analysis of lesions treated with DCB was pre-specified in the clinical investigation plan. In addition, a propensity score model was used, that was built using a logistic regression to model the propensity to use a DCB as a function of the covariates, as detailed in Supplemental Table S1 (*n* = 15 variables). Missing data in the covariates were imputed using a single imputation via Monte Carlo Markov Chain methodology. The model fit was assessed via the c-statistic and the propensity scores before and after matching were plotted to examine the overlap between the groups. A 1:1 matched cohort was constructed using the GREEDY match algorithm. The analysis was performed using SAS version 9.4 (SAS institute, Cary, NC, USA).

## 3. Results

Baseline data have been described previously [2]. In brief, patients were 70.1 ± 10.0 years old on average, 65.5% were male, 68.0% smokers, and 36.9% had diabetes (Table 1). Lesions had a reference vessel diameter of 5.5 ± 0.7 mm and were 125.9 ± 91.0 mm long. The total stented length was 131.2 ± 80.1 mm (Table 2 and Table 3). 

A DCB was used prior to BioMimics 3D stent placement in 23.7% (123/518), and after BioMimics 3D stent deployment, in 26.4% (137/518) of the lesions treated. The three most frequently used DCBs were the Lutonix DCB (34.0%), the In.Pact DCB (32.4%), and the Passeo-18 Lux DCB (14.0%).

**Figure 2:**
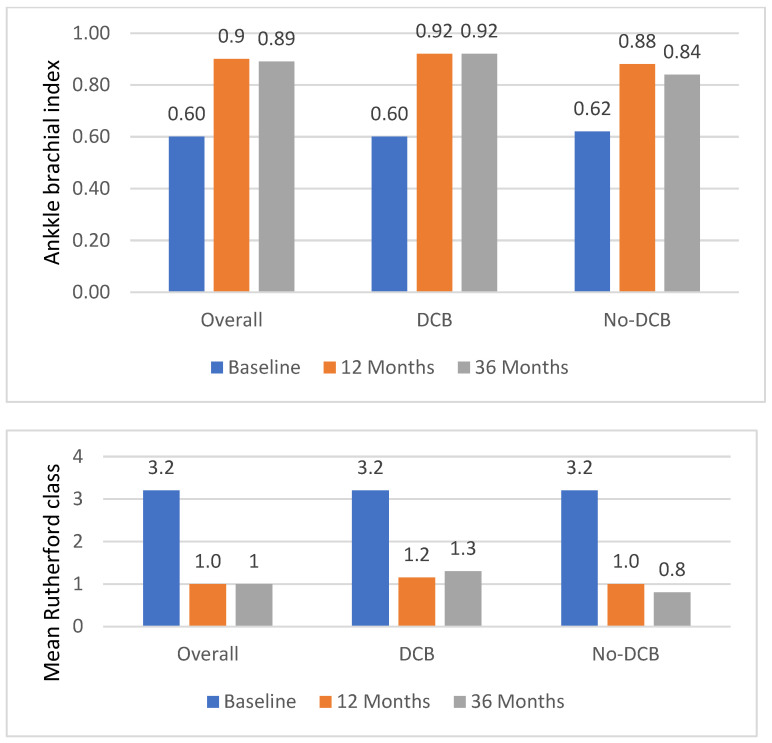
Mean ankle–brachial index and Rutherford class at baseline and follow-up (propensity-matched cohort). Upper panel: there was no statistically significant difference in terms of ankle–brachial index amongst the groups. At baseline, 12 and 36 months, ankle–brachial index data were available for 173, 150 and 115 patients in the DCB group, and for 143, 130 and 66 patients in the no-DCB group. Lower panel: in terms of the Rutherford class, there was a significant difference between the groups at 12 and 36 months (*p* = 0.048 and *p* = 0.0001, respectively). The mean Rutherford class was available for 194, 149 and 141 patients in the DCB group and 193, 141 and 118 in the no-DCB group.

At 3 years, 377 patients returned for their follow-up visit; 72 had died (88.6% follow-up compliance). Per Kaplan–Meier analysis, the overall survival was 85.4%, freedom from major amputation 98.5%, freedom from CD-TLR 78.0%, and site-assessed primary patency rate 70.2% (peak systolic velocity ratio was available for 333 patients at 30 days, and for 301, 237 and 222 patients at 12, 24 and 36 months, respectively) (Table 4). Mean Rutherford class had decreased by 2.0 ± 1.3 classes to 1.00 ± 1.05 (Figure 2).

There was no systematic X-ray assessment to assess stent fractures; nevertheless, four stent fractures were reported (one of which was not confirmed by the clinical events committee).

### Propensity-Matched Cohorts

The propensity-matched cohorts consisted of 195 patients each. Both cohorts were well matched, as shown in Supplemental Figure S1, and Table 1 and Table 2, except for a lower calcification grade rate (grade 4) in the DCB group (7.9% vs. 19.3%, *p* = 0.001). 

The total stent length was 128.3 ± 80.9 mm in the DCB group vs. 132.2 ± 79.2 mm in the no-DCB group, *p* = 0.262 for lesions lengths of 123.6 ± 93.7 mm and 124.7 ± 86.6 mm, *p* = 0.402, respectively (Table 2 and Table 3). In the unmatched cohort, the total stented length was 135.0 ± 85.5 mm vs. 127.7 ± 74.6 mm, *p* = 0.872, and the lesion length was 137.6 ± 99.8 mm vs. 115.0 ± 80.7 mm, *p* = 0.068, respectively. Spot-stenting, which we defined as stented length <80% of lesion length, was performed in 15.9% of lesions in the propensity-matched DCB cohort and 11.3% in the no-DCB cohort, *p* = 0.237, and for 15.3% vs. 11.1%, *p* = 0.239, in the unmatched cohort. 

**Figure 3:**
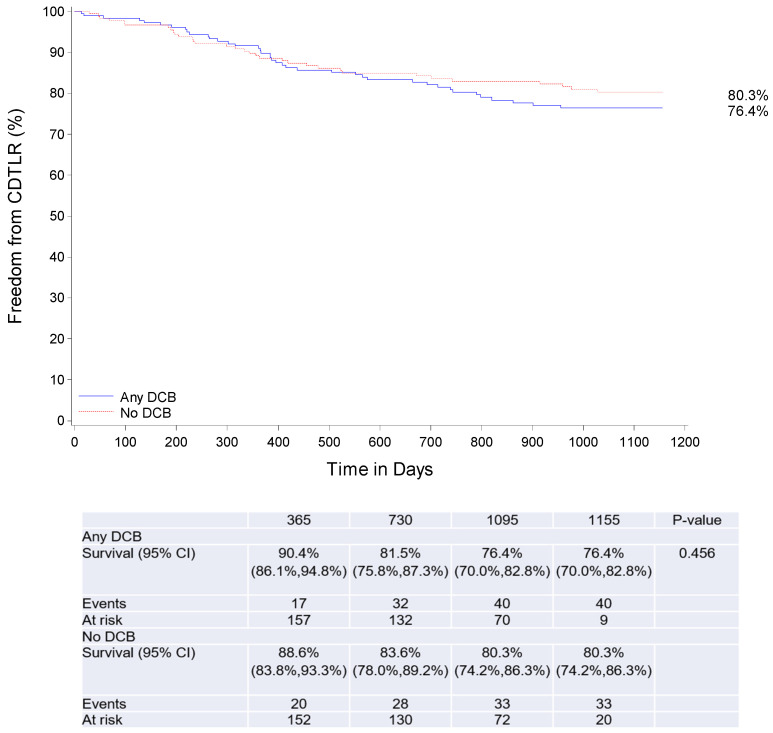
Clinically driven target lesion revascularisation through 3 years (propensity-matched cohorts). CD-TLR—clinically driven target lesion revascularisation, DCB—drug-coated balloon.

At 3 years, 153 patients in the DCB and 145 patients in the no-DCB group completed their follow-up visit, and 22 and 26 patients had died, respectively. There was no statistically significant difference in clinical outcomes. Three-year survival (day 1095) was 87.9% and 85.1% in the DCB and no-DCB propensity-matched cohorts, freedom from major amputation was 99.4% and 97.2%, CD-TLR was 76.4%, and 80.3% and primary patency 68.5% and 74.4%, respectively (Table 4, Figure 3). 

As detailed in Figure 2, the mean baseline Rutherford class was identical amongst the groups (3.2 ± 0.8 in the DCB group and 3.2 ± 1.2 in the no-DCB group), whereas at follow-up, the mean Rutherford class was significantly higher in the DCB group (1.3 ± 1.1 vs. 0.8 ± 1.0, *p* = 0.0001, at 3 years). 

A summary of the unmatched DCB and no-DCB cohort data is provided in Supplemental Tables S2–S5, and a comparison of outcomes in patients that received a DCB before vs. after stent implantation is provided in Supplemental Table S6.

## 4. Discussion

The real-world MIMICS 3D registry showed that treating femoropopliteal lesions using the BioMimics 3D stent results in good 3-year outcomes, whether the stent is used in combination with a DCB or not. 

### 4.1. 3-Year Outcomes in the Overall Cohort

BioMimics 3D is a next-generation stent with a 3D helical centreline that imparts a gentle helical shape to the artery, which allows the stent to shorten, elongate and twist with the artery. Furthermore, the helical geometry induces swirling flow that increases wall shear stress, which has been shown to reduce intimal hyperplasia [5,6]. Moreover, the 3D helical geometry improves the stent’s biomechanical compatibility which reduces vascular injury and the risk of stent fracture, both of which can be the consequence of using traditional straight stents in the mobile femoropopliteal arteries [7]. The stent demonstrated statistically significant better primary patency at 2 years compared to straight stents, and numerically higher freedom from TLR in a randomised controlled trial, where the curves diverged beyond 12 months in favour of the BioMimics 3D stent [4]. 

Likewise, the MIMICS 3D registry showed good outcomes in terms of TLR. Putting the data into perspective, whilst acknowledging that at this stage there are no direct RCTs comparing BioMimics 3D to drug-based devices, the MIMICS 3D TLR rate is similar to that seen in DES and DCB trials, whether in the overall, DCB or no-DCB cohort. In the overall cohort, with a mean lesion length of 125.9 ± 91.0 mm, freedom from CD-TLR at 3 years was slightly lower (78.0%) compared to 87.2% for the Zilver PTX DES (Cook Medical, Bloomington, Indiana) in the Zilver PTX randomized controlled trial, and 85.3% in the MAJESTIC trial using the Eluvia DES (Boston Scientific, Marlborough, MA), but in those trials, lesions were only approximately half as long as those in MIMICS 3D (lesion lengths of 66.4 mm and 70.8 mm, respectively) [8,9]. The Real-PTX randomised controlled trial had slightly longer lesions with 155.5 mm, and a lower freedom from TLR (68.9% for the Zilver PTX stent and 71.3% for DCBs) [10]. The In.Pact Global DCB registry had a similar lesion length (120.9 mm, provisional stenting in 21.2%), and reported 76.9% freedom from TLR at 3 years [11].

### 4.2. BioMimics 3D in Combination with DCB

Nearly half of the lesions in MIMICS 3D were additionally treated with a DCB. This, along with the very good outcomes, warrants further investigations of the effect of additional DCB therapy. 

There are several reasons to consider the combination of bare metal stents and DCB therapy. First, stenting is used as bailout therapy after DCB therapy. This is required in around one quarter of procedures increasing with increased lesion length or when occlusions are treated [10,11,12,13,14,15]. In the IN.PACT Global study, provisional stenting was performed in 36.4% of cases in lesions ≥ 150 mm [13].

Second, the combination of stent plus DCB may be used in the context of spot-stenting. Spot-stenting has the advantage of limiting axial stiffness of the artery, possibly preventing kinking and occlusion, as the vessel (in contrast to DES treatment) is only stented where needed, avoiding “full-metal jackets” [16,17]. With the limitation that we arbitrarily determined spot-stenting as the total stented length ≤ 80% of lesion length, 15% of DCB lesions were categorised as having been treated with spot-stenting.

Third, the combination of a stent with a DCB is attractive as it can address recoil post-DCB and reduce restenosis through the addition of a drug, without the irritation of the drug-releasing excipients. In our series, more than half of the lesions were treated with primary stent placement followed by DCB treatment, predominantly to treat complex lesions, combining the advantages of a stent and DCB. This may be particularly useful in long lesions [18].

If DCB therapy is combined with a stent, it makes sense to use a stent with good long-term outcomes that could extend the period of high patency beyond the initial 12 months when drug-eluting devices might lose their effectiveness [13]. BioMimics 3D might be particularly useful in this scenario as it has prolonged clinical benefits due to its helical centreline, mimicking natural arterial geometry and inducing swirling flow as discussed above. This theoretical advantage has been tested in preclinical bench tests, but also in vivo, when BioMimics 3D demonstrated superior patency rates compared to straight nitinol stents [4]. Moreover, the transition zones which have reduced radial force at the stent edges might reduce edge reactions in the highly mobile and dynamic femoropopliteal region [19]. The purpose of the reduction in radial force is to mitigate a step change in profile at the stent ends, which could otherwise lead to a flow disturbance.

Notably, in our series, CD-TLR and patency rates were good in all subgroups, without a significant difference between DCB and non-DCB-treated lesions, whether in the unmatched or propensity-matched analysis. As for the overall cohort, outcomes were comparable to contemporary DES and DCB therapies. A randomised controlled trial would be desired to allow a direct comparison between the different treatment modalities.

### 4.3. Limitations

Potential limitations include those inherent to observational real-world studies. MIMICS 3D is not randomised, hampering the comparison to other devices and leaving room for bias. Inherent to a registry, patients were treated according to the standard of care. This means that the use of a DCB was left to the discretion of the operator. Furthermore, no routine imaging follow-up was performed, which might have resulted in an underreporting of stent fractures or restenosis. No imaging core laboratory was used, therefore the description of lesion length and patency was left to the study centre. Furthermore, the cut-off value for spot-stenting was arbitrarily selected. Lastly, while the propensity matching aimed to reduce the impact of baseline characteristics, results might still be biased because propensity matching is not as effective as randomisation. 

## 5. Conclusions

The MIMICS 3D registry showed satisfying outcomes in patients treated with the helical centreline BioMimics 3D nitinol stent, which are comparable to those of drug-eluting stents. There was no difference in terms of CD-TLR, whether the stent was used alone or in combination with a DCB. This means that suboptimal results after DCB treatment should not be accepted as the combination of DCB plus BioMimics 3D results in good outcomes, and that DCB, in addition to BioMimics 3D stent treatment, may be an alternative to DES therapy when drug treatment is deemed necessary. Ultimately, a randomised controlled trial is needed to allow for a comparison between the different treatment modalities.

## Figures and Tables

**Table 1:** Baseline characteristics.

	Full Cohort	Propensity-Matched Cohort		
		Any DCB	No DCB	*p*-Value
	N = 507	N = 195	N = 195	
Age (years)	70.1 ± 10.0	70.4 ± 9.6	69.7 ± 10.0	0.419
Male	332/507 (65.5%)	129/195 (66.2%)	131/195 (67.2%)	0.915
CVA or TIA	57/507 (11.2%)	20/195 (10.3%)	22/195 (11.3%)	0.870
Hypertension	434/507 (85.6%)	172/195 (88.2%)	158/195 (81.0%)	0.067
Hypercholesterolemia/dyslipidemia	324/507 (63.9%)	131/195 (67.2%)	129/195 (66.2%)	0.915
Coronary artery disease	51/507 (10.1%)	19/195 (9.7%)	23/195 (11.8%)	0.625
Smoking	345/507 (68.0%)	130/195 (66.7%)	136/195 (69.7%)	0.587
Current	191/507 (37.7%)	71/195 (36.4%)	76/195 (39.0%)	0.676
Diabetes mellitus	187/507 (36.9%)	76/195 (39.0%)	73/195 (37.4%)	0.835
Insulin	82/507 (16.2%)	24/195 (12.3%)	34/195 (17.4%)	0.200
Renal insufficiency	42/507 (8.3%)	13/195 (6.7%)	17/195 (8.7%)	0.569
Dialysis	10/507 (2.0%)	3/195 (1.5%)	5/195 (2.6%)	0.724
Non-healing wound on the target limb	73/507 (14.4%)	22/195 (11.3%)	30/195 (15.4%)	0.297

Data are displayed as the mean ± SD or n/N (%). CVA—cerebrovascular accident, TIA—transient ischemic attack.

**Table 2:** Lesion characteristics.

	Full Cohort	Propensity-Matched Cohort		
		Any DCB	No DCB	*p*-Value
	N = 518 Lesions	N = 202 Lesions	N = 198 Lesions	
Target Lesion Type				
De novo	467/518 (90.2%)	183/202 (90.6%)	177/198 (89.4%)	0.741
Restenotic	51/518 (9.8%)	19/202 (9.4%)	21/198 (10.6%)	0.741
Maximal RVD (mm)	5.5 ± 0.7	5.4 ± 0.6 (202)	5.4 ± 0.7 (198)	0.760
Lesion length (mm)	125.9 ± 91.0	123.6 ± 93.7 (202)	124.7 ± 86.6 (198)	0.402
Diameter stenosis (%)	94.6 ± 8.0	95.2 ± 7.2 (202)	94.2 ± 8.1 (198)	0.318
Occlusion	294/518 (56.8%)	119/202 (58.9%)	110/198 (55.6%)	0.545
Calcification				
Grade 0 (no visible calcium)	91/516 (17.6%)	41/202 (20.3%)	29/197 (14.7%)	0.150
Grade 1 (unilateral, <5 cm)	152/516 (29.5%)	68/202 (33.7%)	53/197 (26.9%)	0.157
Grade 2 (unilateral, ≥5 cm)	125/516 (24.4%)	48/202 (23.8%)	48/197 (24.4%)	0.907
Grade 3 (bilateral, <5 cm)	76/516 (14.7%)	29/202 (14.4%)	29/197 (14.7%)	1.0000
Grade 4 (bilateral, ≥5 cm)	71/516 (13.8%)	16/202 (7.9%)	38/197 (19.3%)	0.001

Data are displayed as the mean ± SD or n/N (%). Data are site-assessed. RVD—reference vessel diameter.

**Table 3:** Procedural characteristics.

	Full Cohort	Propensity-Matched Cohort		
		Any DCB	No DCB	*p*-Value
	N = 507 Patients	N = 195 Patients	N = 195 Patients	
	N = 518 Lesions	N = 202 Lesions	N = 198 Lesions	
Procedure time (min)	74.6 ± 48.8	74.3 ± 51.0 (194)	74.2 ± 50.3 (194)	0.621
Total fluoroscopy time (min)	14.9 ± 11.1	15.4 ± 10.8	14.6 ± 12.3	0.09
Total amount of contrast (mL)	111.8 ± 60.2	135.7 ± 61.2 (194)	90.9 ± 49.4 (193)	<0.0001
Guide wire size (inch)				
0.035	269/507 (53.1%)	92/195 (47.2%)	122/195 (62.6%)	0.003
0.018	209/507 (41.2%)	88/195 (45.1%)	69/195 (35.4%)	0.063
0.014	29/507 (5.7%)	15/195 (7.7%)	4/195 (2.1%)	0.017
Number of BioMimics stent deployed				
1	395/518 (76.3%)	153/202 (75.7%)	148/198 (74.7%)	0.908
2	96/518 (18.5%)	38/202 (18.8%)	41/198 (20.7%)	0.707
3	19/518 (3.7%)	8/202 (4.0%)	6/198 (3.0%)	0.787
4	8/518 (1.5%)	3/202 (1.5%)	3/198 (1.5%)	1.000
Total stented length	131.2 ± 80.1	128.3 ± 80.9 (202)	132.2 ± 79.2 (198)	0.262
Spot-stenting (in any lesion) *	74/507 (14.6%)	31/195 (15.9%)	22/195 (11.3%)	0.237
Total stent length spot-stenting (mm)	114.0 ± 52.1	106.8 ± 56.1	117.7 ± 52.4	
No patent infrapopliteal vessel	22/507 (4.3%)	7/195 (3.6%)	11/195 (5.6%)	0.470
PTA balloon				
Pre-dilatation	454/518 (87.6%)	188/202 (93.1%)	167/198 (84.3%)	0.007
Post-dilatation	353/518 (68.1%)	77/202 (38.1%)	185/198 (93.4%)	<0.0001
DCB				
Pre-BioMimics stent placement	123/518 (23.7%)	94/202 (46.5%)	-	-
Post-BioMimics stent placement	137/518 (26.4%)	113/202 (55.9%)	-	-

Data are displayed as the mean ± SD or n/N(%). * stented length <20% of lesion length. DCB—drug-coated balloon, PTA—percutaneous transluminal angioplasty.

**Table 4:** Clinical outcomes per Kaplan–Meier analysis.

	Full Cohort	Propensity-Matched Cohort		
		Any DCB	No DCB	Log-Rank
				*p*-Value
**12-month follow-up (day 365)**				-
Survival	93.9% [91.8; 96.1]	94.1% [90.8; 97.5]	95.6% [92.6; 98.6]	-
Freedom from major amputation	98.5% [97.3; 99.6]	99.4% [98.3; 100]	97.2% [94.8; 99.6]	-
Freedom from CD-TLR	90.0% [87.3; 92.8]	90.4% [86.1; 94.8]	88.6% [83.8; 93.3]	-
Primary patency	87.2% [84.1; 90.2]	89.9% [85.4; 94.3]	86.3% [81.2; 91.4%]	-
**36-month follow-up (day 1095)**				
Survival	85.4% [82.2; 88.7]	87.9% [83.1; 92.6]	85.1% [79.7; 90.4]	0.411
Freedom from major amputation	98.5% [97.3; 99.6]	99.4% [98.3; 100]	97.2% [94.8; 99.6]	0.097
Freedom from CD-TLR	78.0% [74.1; 81.9]	76.4% [70.0; 82.8]	80.3% [74.2; 86.3]	0.456
Primary patency	70.2% [65.8; 74.6]	68.5% [61.3; 75.7]	74.4% [67.6; 81.2]	0.287

Data are displayed as the Kaplan–Meier estimate [95%CI]. CD-TLR—clinically driven TLR.

## Data Availability

The data associated with the paper are not publicly available, but are available from the corresponding author on reasonable request.

## Supplementary Materials

The following supporting information can be downloaded at: https://www.mdpi.com/article/10.3390/jcdd10030126/s1, Table S1: Logistic regression results for a drug-coated balloon vs. no drug-coated balloon (c-statistic = 0.68); Figure S1: Propensity score distribution in the full dataset prior to matching (left panel) and in the matched cohort (right panel); Table S2: Baseline characteristics (unmatched cohorts); Table S3: Lesion characteristics (unmatched cohort); Table S4: Procedural characteristics (unmatched cohorts); Table S5: Three-year clinical outcomes per Kaplan–Meier estimate (unmatched cohorts); Table S6: Three-year clinical outcomes per Kaplan-Meier estimates in patients that received DCB therapy before and after stent placement.
