# Supplementary material for: BioMimics 3D Stent in Femoropopliteal Lesions: 3-Year Outcomes with Propensity Matching for Drug-Coated Balloons
Source: J Cardiovasc Dev Dis. 2023 Mar 16;10(3):126. doi: 10.3390/jcdd10030126 (PMC10051351; doi:10.3390/jcdd10030126)
Supplement: Supplementary file 1 [file jcdd-10-00126-s001.zip › jcdd-2281958-supple.docx]

**Drug-coated balloons in combination with a helical centreline stent in femoropopliteal lesions: 3-year outcomes from the MIMICS-3D registry including propensity matching**

**Supplemental Table S1: Logistic regression results for drug-coated balloon versus no drug-coated balloon (c-statistic=0.68)**

|  | **Odds Ratio** | **95% CI** | | **P-value** |
| --- | --- | --- | --- | --- |
| Intercept |  |  | | 0.020 |
| Age (per 10 Years) | 0.81 | 0.65 | 1.01 | 0.057 |
| Number of Stents | 0.89 | 0.61 | 1.31 | 0.562 |
| Female | 1.29 | 0.86 | 1.93 | 0.226 |
| Diabetes | 1.03 | 0.70 | 1.54 | 0.868 |
| Smoking Current | 1.01 | 0.65 | 1.57 | 0.951 |
| Rutherford | 1.10 | 0.91 | 1.34 | 0.318 |
| Hypercholesterolemia | 2.22 | 1.49 | 3.30 | <0.001 |
| Renal Failure | 0.47 | 0.23 | 0.97 | 0.040 |
| Lesion Length>140 | 1.61 | 0.95 | 2.73 | 0.079 |
| RVD>5 | 1.66 | 1.12 | 2.46 | 0.011 |
| Total Occlusion | 0.66 | 0.36 | 1.23 | 0.191 |
| Tibial Runoff 2+ | 1.10 | 0.69 | 1.74 | 0.691 |
| Calcification | 0.84 | 0.57 | 1.23 | 0.366 |
| DeNovo | 1.27 | 0.67 | 2.39 | 0.461 |
| % Diameter stenosis (per 10%) | 1.70 | 0.95 | 2.73 | 0.009 |

RVD-reference vessel diameter

**Supplemental Figure S1: Propensity Score distribution in the full dataset prior to matching (left panel) and in the matched cohort (right panel)**


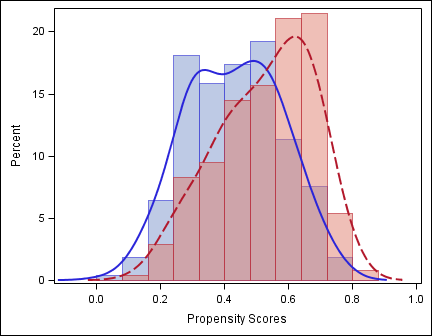

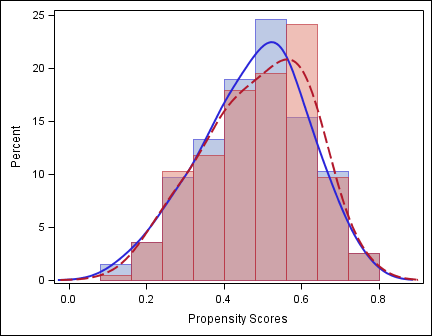


The full dataset includes 507 patients and the matched cohort 195 each

**Supplemental Table S2: Baseline characteristics (unmatched cohorts)**

|  | **DCB**  **N=242** | **No DCB**  **N=265** | **P-value** |
| --- | --- | --- | --- |
| Age (years) | 69.2 ± 10.0 | 70.9 ± 9.9 | 0.091 |
| Male | 154 / 242 (63.6%) | 178 / 265 (67.2%) | 0.455 |
| Hypertension | 216 / 242 (89.3%) | 218 / 265 (82.3%) | 0.031 |
| Hypercholesterolemia / dyslipidemia | 177 / 242 (73.1%) | 147 / 265 (55.5%) | <.0001 |
| Coronary artery disease | 22 / 242 (9.1%) | 29 / 265 (10.9%) | 0.555 |
| Smoking | 167 / 242 (69.0%) | 178 / 265 (67.2%) | 0.703 |
| Diabetes mellitus | 90 / 242 (37.2%) | 97 / 265 (36.6%) | 0.927 |
| Insulin | 31 / 242 (12.8%) | 51 / 265 (19.2%) | 0.054 |
| Non-healing wound on the target limb | 29 / 242 (12.0%) | 44 / 265 (16.6%) | 0.164 |

Data are displayed as mean ±SD or n/N (%)

**Supplemental Table S3: Lesion characteristics (unmatched cohort)**

|  | **Any DCB**  **N=250** | **No DCB**  **N=268** | **P-value** |
| --- | --- | --- | --- |
| Target Lesion Type |  |  |  |
| De novo | 228 / 250 (91.2%) | 239 / 268 (89.2%) | 0.464 |
| Restenotic | 22 / 250 (8.8%) | 29 / 268 (10.8%) | 0.464 |
| Maximal RVD (mm) | 5.5 ± 0.6 | 5.4 ± 0.7 | 0.083 |
| Lesion length (mm) | 137.6 ± 99.8 (250) | 115.0 ± 80.7 (268) | 0.068 |
| Diameter stenosis (%) | 95.9 ± 6.8 (250) | 93.5 ± 8.9 (268) | 0.004 |
| Occlusion | 155 / 250 (62.0%) | 139 / 268 (51.9%) | 0.021 |
| Calcification |  |  |  |
| Grade 0 (no visible calcium) | 52 / 250 (20.8%) | 39 / 266 (14.7%) | 0.083 |
| Grade 1 (unilateral, < 5cm) | 84 / 250 (33.6%) | 68 / 266 (25.6%) | 0.053 |
| Grade 2 (unilateral, ≥ 5cm) | 58 / 250 (23.2%) | 68 / 266 (25.6%) | 0.540 |
| Grade 3 (bilateral, < 5cm) | 33 / 250 (13.2%) | 43 / 266 (16.2%) | 0.385 |
| Grade 4 (bilateral, ≥ 5cm) | 23 / 250 (9.2%) | 48 / 266 (18.0%) | 0.005 |

Data are displayed as mean ±SD or n/N (%). Data are site-assessed. RVD-Reference vessel diameter

**Supplemental Table S4: Procedural characteristics (unmatched cohorts)**

|  | **Any DCB**  **N=242 patients**  **N=250 lesions** | **No DCB**  **N=265 patients**  **N=268 lesions** | **P-value** |
| --- | --- | --- | --- |
| Procedure time (min) | 75.6 ± 48.5 | 73.6 ± 49.1 | 0.207 |
| Total Fluoroscopy time | 15.9 ± 11.0 | 13.9 ± 11.1 | 0.002 |
| Total Amount of contrast (mL) | 136.0 ± 61.7 | 89.4 ± 49.2 | <0.0001 |
| Guide Wire size (inch) |  |  |  |
| 0.035 | 108 / 242 (44.6%) | 161 / 265 (60.8%) | 0.0004 |
| 0.018 | 112 / 242 (46.3%) | 97 / 265 (36.6%) | 0.030 |
| 0.014 | 22 / 242 (9.1%) | 7 / 265 (2.6%) | 0.002 |
| Total stented length | 135.0 ± 85.5 | 127.7 ± 74.6 | 0.872 |
| Spot-stenting (in any lesion)*  Total stented length spot-stenting (mm) | 51 / 250 (20.4%)  112.2 ± 52.9 | 23 / 268 (8.6%)  118.0 ± 51.2 | 0.0001  0.456 |
| No patent infrapopliteal vessel | 8 / 242 (3.3%) | 14 / 265 (5.3%) | 0.383 |
| PTA balloon |  |  |  |
| Pre-dilatation | 233 / 250 (93.2%) | 221 / 268 (82.5%) | 0.0003 |
| Post-dilatation | 101 / 250 (40.4%) | 252 / 268 (94.0%) | <.0001 |
| DCB |  |  |  |
| Pre-BioMimics stent placement | 123 / 250 (49.2%) | - | - |
| Post-BioMimics stent placement | 137 / 250 (54.8%) | - | - |

Data are displayed as mean ± SD or n/N(%). *stented length <20% of lesion length. DCB-drug-coated balloon, PTA-percutaneous transluminal angioplasty

**Supplemental Table S5: 3-year Clinical Outcomes per Kaplan-Meier estimate (unmatched cohorts)**

|  | **Any DCB**  **N=242 patients** | **No DCB**  **N=265 patients** | **P-value** |
| --- | --- | --- | --- |
| Overall survival | 89.4% | 81.6% | 0.019 |
| Freedom from major amputation | 99.5% | 97.4% | 0.066 |
| Freedom from CD-TLR | 76.4% | 79.6% | 0.479 |
| Primary patency | 66.8% | 73.6% | 0.143 |

Data are displayed as Kaplan-Meier estimates. CD-TLR- clinically-driven TLR, DCB-drug-coated balloon

**Supplemental Table S6: 3-year Clinical Outcomes per Kaplan-Meier estimate in patients that received DCB-therapy before or after stent placement**

|  | **Pre-dilatation DCB**  **N=118 patients** | **Post-dilatation DCB**  **N=135 patients** |
| --- | --- | --- |
| Overall survival | 90.8% | 88.3% |
| Freedom from major amputation | 99.1% | 100% |
| Freedom from CD-TLR | 76.7% | 76.4% |
| Primary patency | 68.6% | 65.5% |
